# Prevalence of hepatitis B and C infection in persons living with HIV enrolled in care in Rwanda

**DOI:** 10.1186/s12879-017-2422-9

**Published:** 2017-05-02

**Authors:** Justine Umutesi, Bryony Simmons, Jean D. Makuza, Donatha Dushimiyimana, Aimable Mbituyumuremyi, Jean Marie Uwimana, Nathan Ford, Edward J. Mills, Sabin Nsanzimana

**Affiliations:** 1 HIV/AIDS & STIs Diseases Division, Rwanda Biomedical Centre, Kigali, Rwanda; 2 Clinton Health Access Initiative, Kigali, Rwanda; 3 Centre for Infectious Disease Epidemiology and Research, University of Cape Town, Cape Town, South Africa; 4 University of Rwanda, Kigali, Rwanda; 5 Basel Institute for Clinical Epidemiology and Biostatistics, University Hospital Basel, Basel, Switzerland; 6 Swiss Tropical and Public Health Institute, University of Basel, Basel, Switzerland

**Keywords:** HIV, Hepatitis, Co-infection, Prevalence

## Abstract

**Background:**

Hepatitis B (HBV) and C (HCV) are important causes of morbidity and mortality in people living with human immunodeficiency virus (HIV). The burden of these co-infections in sub-Saharan Africa is still unclear. We estimated the prevalence of the hepatitis B surface antigen (HBsAg) and hepatitis C antibody (HCVAb) among HIV-infected individuals in Rwanda and identified factors associated with infection.

**Methods:**

Between January 2016 and June 2016, we performed systematic screening for HBsAg and HCVAb among HIV-positive individuals enrolled at public and private HIV facilities across Rwanda. Results were analyzed to determine marker prevalence and variability by demographic factors.

**Results:**

Overall, among 117,258 individuals tested, the prevalence of HBsAg and HCVAb was 4.3% (95% confidence interval [CI] (4.2–4.4) and 4.6% (95% CI 4.5–4.7) respectively; 182 (0.2%) HIV+ individuals were co-infected with HBsAg and HCVAb. Prevalence was higher in males (HBsAg, 5.4% [5.1–5.6] vs. 3.7% [3.5–3.8]; HCVAb, 5.0% [4.8–5.2] vs. 4.4% [4.3–4.6]) and increased with age; HCVAb prevalence was significantly higher in people aged ≥65 years (17.8% [16.4–19.2]). Prevalence varied geographically.

**Conclusion:**

HBV and HCV co-infections are common among HIV-infected individuals in Rwanda. It is important that viral hepatitis prevention and treatment activities are scaled-up to control further transmission and reduce the burden in this population. Particular efforts should be made to conduct targeted screening of males and the older population. Further assessment is required to determine rates of HBV and HCV chronicity among HIV-infected individuals and identify effective strategies to link individuals to care and treatment.

## Background

Viral hepatitis has emerged as an important public health issue globally, characterised by high prevalence, a high burden of morbidity and mortality, and suboptimal diagnosis and management approaches [1, 2].

Viral hepatitis disproportionately affects persons living with human immunodeficiency virus (HIV) [3, 4]. While varying in their transmission efficiency, HIV, hepatitis B (HBV), and hepatitis C (HCV) share common routes of transmission, and as such, prevalence is generally higher in HIV-infected individuals [3–5]. To compound this, HIV infection accelerates the natural course of HBV and HCV, promoting faster progression to chronicity, liver fibrosis, and malignancy [4, 6, 7]. As antiretroviral therapy (ART) programmes have matured and people with HIV survive longer, viral hepatitis has become a major cause of death in HIV-infected individuals [8].

Sub-Saharan Africa (SSA) has the highest burden of infectious diseases and remains the epicentre of the global HIV epidemic [9, 10]. Despite this, epidemiological data for HBV and HCV are lacking due to limited diagnostic capacity and a lack of prioritisation in public health programmes. Available data suggest widely variable rates of HBV and HCV infection in HIV-infected populations across SSA. In one meta-analysis, HIV/HBV frequency in SSA varied from 0% to 28.4% (median 7.8%) [11]. Two recent systematic reviews suggest an overall HCV prevalence of 3% among people living with HIV in SSA with significant regional variations (0–55.9%) [3, 12].

Data from Rwanda is limited. Among 402 HIV-infected, ART naïve individuals recruited from a major Kigali-based urban healthcare facility, a 5.2% hepatitis B surface antigen (HBsAg) and a 5.7% hepatitis C antibody (HCVAb) prevalence were reported [13]. The HBsAg and HCVAb prevalence were 2.4% and 4.9%, respectively, in 82 samples retrospectively analysed from HIV-positive pregnant women in 2004 [14]. A systematic review estimated the prevalence of HCVAb in persons living with HIV was estimated to be less than 3% in Rwanda [3]. Given the paucity of epidemiological data from robust studies, there is a need for nationally representative estimates to ascertain the scope of the viral hepatitis burden among HIV-positive individuals in Rwanda.

Advances in prevention and treatment have facilitated the development of public health programmes to control viral hepatitis in resource limited settings [15, 16]. Rwanda has been one of the first countries in SSA to establish a viral hepatitis control programme. Initiated in 2012 and building on the existing HIV infrastructure, the programme was intensified in 2015 with the arrival of new efficacious treatments for HCV [17]. As part of routine programme implementation, an initiative to screen all HIV-positive individuals enrolled into care for key markers for HBV and HCV was implemented from January to June 2016, reaching approximately 120,000 HIV-infected individuals nationwide.

The present study set out to characterise the prevalence and characteristics of HBsAg and HCVAb in HIV-positive individuals in Rwanda using data collected through this screening programme. Our data provide a picture of HBV and HCV co-infection with HIV in a setting where resources for screening, diagnosis, and treatment are limited. Findings will be fundamental for informing future policy and decision-making in Rwanda.

## Methods

### Study objectives

The primary objective of this study was to quantify the prevalence of HBsAg and HCVAb in HIV-positive individuals enrolled into care using data collected during a national screening activity. Secondary objectives were to analyse data by subgroups.

### Data collection

Data were obtained from the nationally representative electronic database compiled throughout the screening activity conducted between January and June 2016. The programme targeted all individuals with HIV nationwide, enrolling individuals at all public and private health facilities providing HIV services across Rwanda (approximately 500 facilities). Screening was offered free-of-charge to all HIV-positive individuals enrolled in care at a routine appointment, regardless of ART status. HIV-positive individuals are followed up monthly at health facilities and as such, all eligible individuals had the opportunity to undergo screening during the implementation period. All HIV-positive individuals screened were included in the database which captures demographic data, HIV infection and disease characteristics, and HBV and HCV clinical test results.

In brief, testing procedures were as follows: individuals were invited for screening during a routine HIV appointment, specimens were collected at the site of enrolment, and samples transported to a testing facility. Testing was performed at 13 sites across Rwanda using Murex enzyme-linked immunosorbent assays (ELISA) for HBsAg (version 3.0) and HCVAb (version 4.0; DiaSorin S.p.A, Italy). All testing was supervised by a team of laboratory technicians from the National Reference Laboratory.

The database was de-identified for the present study and no persons involved in the analysis of data were able to access the linked database. For comparison, aggregate population data for HIV-infected individuals were obtained from the Rwanda national HIV health record system (TRACnet) and were correct as of June 2016.

### Statistical methods & data analysis

Sample weighting was applied in all analyses to ensure results were nationally representative of people with HIV enrolled into care in Rwanda. The post-stratification weights were calculated using known age, gender, and ART distributions. All continuous variables were categorised for analysis based on appropriate thresholds.

Descriptive statistics were performed for all variables. National prevalence estimates for HBV (HBsAg) and HCV (HCVAb) were calculated and presented with the corresponding 95% confidence intervals (95% CIs). HBsAg and HCVAb prevalence by demographic and HIV characteristics were determined; bivariate associations were tested using Pearson’s chi-square tests. All analyses were performed using Stata software (version 12.0; StataCorp, Texas, USA).

### Ethics

The routinely collected programme data analysed for this study are maintained by the Rwanda Biomedical Centre (RBC), Division of HIV/AIDS, STIs and Other Blood Borne Infections; the ethical procedures for the collection of these data are governed by the Medical Research Council of Rwanda. Secondary analyses of routinely collected data are exempt by the Rwanda Ethics Committee for routine program implementation activities conducted by the RBC, as was the case in this analysis.

## Results

### Testing coverage and population characteristics

A total of 117,258 HIV-positive individuals were screened between January and June 2016 and were included in the present analysis. This corresponds to a coverage level of 64.9% of the total HIV population enrolled to care at facilities across Rwanda. The characteristics of the screened population did not differ significantly from the overall HIV-positive population enrolled to care.

Table 1 describes the socio-demographic and HIV characteristics of the included individuals. The majority of the population were female (65.1%), aged 35–54 years (56.0%), and lived in rural areas (75.0%). Most individuals were enrolled at the health centre or clinic level (84.3%); 15.7% were enrolled at the tertiary hospital level, inclusive of district, provincial, and referral hospitals. Over 90% of individuals were on ART at the time of screening.

**Table 1:** Baseline characteristics and prevalence of HBsAg and HCVAb by demographics and HIV characteristics

	HBsAg (*n* = 114,040)			HCVAb (*n* = 116,868)		
	Total (%)	Positive (95% CI)	*p*-value^a^	Total (%)	Positive (95% CI)	*p*-value^a^
Overall						
All	114,040	4.3 (4.2–4.4)		116,868	4.6 (4.5–4.7)	
Demographic factors						
Age			**<0.005**			**<0.005**
< 15	4917 (4.4)	2.1 (1.7–2.6)		5062 (4.4)	1.7 (1.3–2.1)	
15–24	7409 (6.6)	4.0 (3.5–4.4)		7616 (6.6)	2.1 (1.7–2.4)	
25–34	21,287 (19.0)	4.2 (3.9–4.5)		21,838 (19.0)	3.1 (2.9–3.4)	
35–44	34,113 (30.4)	4.8 (4.5–5.0)		34,971 (30.5)	3.8 (3.5–4.0)	
45–54	28,719 (25.6)	4.5 (4.2–4.7)		29,371 (25.6)	4.7 (4.5–5.0)	
55–64	12,582 (11.2)	4.0 (3.6–4.4)		12,871 (11.2)	8.8 (8.3–9.3)	
≥ 65	3037 (2.7)	3.9 (3.2–4.6)		3098 (2.7)	17.8 (16.4–19.2)	
Gender			**<0.005**			**<0.005**
Female	74,141 (65.1)	3.7 (3.5–3.8)		75,946 (65.1)	4.4 (4.3–4.6)	
Male	39,751 (34.9)	5.4 (5.1–5.6)		40,774 (34.9)	5.0 (4.8–5.2)	
Enrolment facility			**0.031**			**0.013**
Health center	92,576 (84.2)	4.2 (4.0–4.3)		94,991 (84.3)	4.5 (4.4–4.7)	
Hospital	17,346 (15.8)	4.5 (4.2–4.9)		17,715 (15.7)	5.0 (4.7–5.3)	
Geographical factors						
Province			**<0.005**			**<0.005**
Kigali	19,135 (16.8)	5.0 (4.7–5.3)		19,413 (16.6)	4.0 (3.7–4.2)	
East	23,404 (20.5)	5.5 (5.2–5.8)		25,874 (22.1)	5.2 (4.9–5.5)	
North	16,094 (14.1)	4.2 (3.9–4.6)		16,114 (13.8)	4.2 (3.9–4.6)	
South	27,368 (24.0)	3.6 (3.4–3.9)		27,367 (23.4)	5.9 (5.6–6.2)	
West	28,027 (24.6)	3.5 (3.3–3.7)		28,086 (24.0)	3.5 (3.3–3.8)	
Residence^b^			**<0.005**			**0.013**
Rural	73,820 (74.7)	4.0 (3.9–4.2)		76,070 (75.1)	4.7 (4.5–4.8)	
Urban	24,975 (25.3)	4.9 (4.7–5.2)		25,198 (24.9)	4.3 (4.0–4.6)	
HIV characteristics						
ART category			0.214			**<0.005**
On ART	98,974 (91.6)	4.3 (4.2–4.5)		101,510 (91.5)	4.4 (4.3–4.5)	
Pre ART	9119 (8.4)	4.1 (3.6–4.5)		9381 (8.5)	6.6 (6.1–7.1)	
CD4 count^b^			**<0.005**			**<0.005**
≤ 500	34,698 (41.2)	4.9 (4.7–5.1)		35,339 (41.2)	5.0 (4.8–5.3)	
> 500	49,469 (58.8)	3.7 (3.6–3.9)		50,497 (58.8)	4.4 (4.2–4.6)	
HIV viral load^b^			**<0.005**			0.433
≤ 1000	66,272 (95.1)	3.9 (3.7–4.1)		67,424 (95.1)	4.3 (4.1–4.5)	
> 1000	3406 (4.9)	5.5 (4.6–6.4)		3486 (4.9)	4.6 (3.8–5.4)	

Results are proportion positive (95% confidence interval); Sampling weights have been applied

^a^Reported are Chi-Square *p*-values; statistically significant *p*-values are in bold (*p* < 0.05)

^b^Missing responses ≥10%

### Prevalence

The overall HBsAg and HCVAb prevalence in the HIV-infected population and the prevalence by socio-demographic and HIV characteristics are shown in Table 1.

### HBV prevalence

**Fig. 1:**
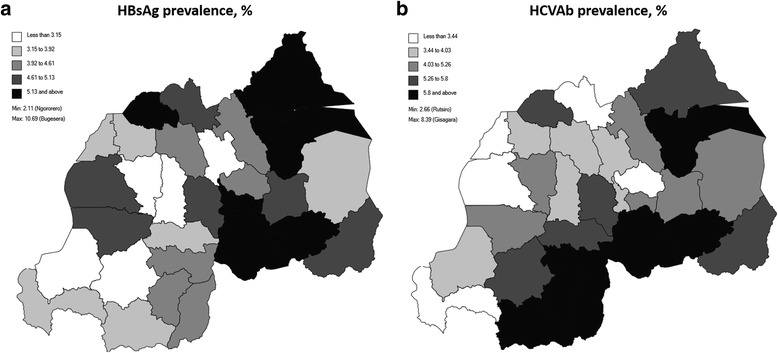
Prevalence of **a** HBsAg and **b** HCVAb by District, Rwanda

In total, HBsAg result were available for 114,040/117,258 (97.3%) individuals included in the database. Overall, 4876/114,040 individuals were HBsAg positive. The unadjusted and adjusted prevalence of HBsAg were equal at 4.3% (95% CI 4.2–4.4%). Prevalence was significantly higher in males compared with females (5.4% [95% CI 5.1–5.6%] vs. 3.7% [95% CI 3.5–3.8%]). Prevalence varied by age group and was lowest in individuals less than 15 years of age (2.1% [95% CI 1.7–2.6%]). HBsAg prevalence was slightly higher at hospital enrolment compared with enrolment at primary care level (health centres and clinics). By geographical region, prevalence was highest in the Eastern province (5.5% [95% CI 5.2–5.8%]) and in the City of Kigali (5.0% [95% CI 4.7–5.3%]); prevalence was significantly higher in urban areas compared with rural areas (4.9% [95% CI 4.7–5.2%] vs. 4.0% [95% CI 3.9–4.2%]). Prevalence by district is shown in (Fig. 1); prevalence varied from 10.7% (Bugesera) down to 2.1% (Ngororero).

### HCV prevalence

In total, HCVAb results were available for 116,868/117,258 (99.7%) individuals included on the database. For HCVAb, 5470/116,868 individuals were found to be positive. The unadjusted prevalence was 4.7% (95% CI 4.5–4.8%) and the adjusted prevalence was 4.6% (95% CI 4.5–4.7%). Like HBsAg, prevalence was higher in males than females (5.0% [95% CI 4.8–5.2%] vs. 4.4% [4.3–4.6%]). Prevalence increased with age, and was significantly higher in individuals aged 55–64 (8.8% [95% CI 8.3–9.3%]) and those aged 65 or over (17.8% [95% CI 16.4–19.2%]). Prevalence was slightly higher at hospitals compared with primary care facilities (5.0% [95% CI 4.7–5.3%] vs. 4.5% [95% CI 4.4–4.7%]). HCVAb prevalence was highest in the Southern (5.9% [95% CI 5.6–6.2%]) and Eastern (5.2% [95% CI 4.9–5.5%]) provinces of Rwanda. In contrast to HBsAg prevalence, HCVAb prevalence was higher in rural areas (4.7% [95% CI 4.5–4.8%]) than urban areas (4.3% [95% CI 4.0–4.6]); however, this difference was not significant. Prevalence of HCVAb by district is shown in Fig. 1; HCVAb prevalence varied from 8.4% (Gisagara) to 2.7% (Rutsiro) and was generally higher in districts bordering other countries.

### Co-infection

Of 117,258 individuals included in the database, 113,681 (96.9%) had results for both HBsAg and HCVAb. A total of 182/113,681 individuals were coinfected with HBsAg and HCVAb, corresponding to an adjusted co-infection prevalence of 0.2% (95% CI 0.1–0.2%).

## Discussion

Advances in treatment and the benefits of early detection have validated the scale-up of viral hepatitis screening efforts in resource-limited settings [15, 18]. Accordingly, a national programme was recently conducted in Rwanda to screen all persons with HIV for key HBV and HCV markers. The high screening uptake, reaching almost 120,000 individuals, demonstrates that large regional or national screening programmes for viral hepatitis are feasible among HIV population in resource-limited settings. Given the limited resources available for population screening efforts, the present study aimed to analyse the results to contribute towards the understanding of the viral hepatitis co-infection burden in Rwanda and to guide prioritisation for testing of key at-risk and geographic populations.

Our study estimates the prevalence of HBsAg among HIV-infected individuals to be 4.3%. This is consistent with other studies conducted in Rwanda and should be considered a robust estimate given the large sample size [13, 14]. Due to the cross-sectional nature of this study, we were unable to verify the persistence of HBsAg over time; HBsAg is a good proxy for chronicity and we assume that HBsAg-positivity represents chronic infection. In accordance with the Rwanda viral hepatitis treatment guidelines, all individuals positive for HBsAg will be initiated on tenofovir-based ART.

The epidemiology of HBsAg is characterised by a higher prevalence in males, whereby males have an almost 50% greater risk of becoming HBV infected. This finding is in concordance with other studies conducted in SSA [19]. HIV/HBV coinfection patterns vary geographically from 2.1% up to 10.7% across the 30 districts of Rwanda.

Of note, the HBsAg prevalence is lowest in children below 15 years, motivated by the roll-out of HBV vaccination activities since 2002. Despite this decrease, our findings indicate that strengthening of vaccination activities is required. At present, the HBV birth-dose vaccination, administered within 24 h of birth is not implemented and should be considered as a strategy to reduce perinatal transmission [18]. Further, HBV vaccination in HIV-positive populations may provide a moderately lower response rate than in the general population, and as such, implementing polices with 1) specific immunisation algorithms for HIV-infected/exposed children and 2) vaccine response monitoring should be explored [19–21].

We found an HCVAb seroprevalence of 4.6% among HIV-infected individuals, marginally lower than that observed in previous studies (4.9–5.6%) [13, 14]. Consistent with findings from other SSA populations, HCVAb epidemiology is characterised by a higher prevalence in males, and by an increasing prevalence with the increase in age [22–25]. Cumulative exposure may explain the association between HCVAb prevalence and older age. However, given the strikingly higher prevalence in individuals over 55 years, it is likely the association is impacted by a cohort effect of patients infected many years ago. Our findings indicate that one-time screening of individuals aged over 55 should be recommended. Birth-cohort screening is in practice or has been evaluated in various other settings and has largely been identified as a cost-effective initiative [26–29].

HCVAb prevalence varies geographically from 2.7% to 8.4% across Rwanda’s 30 districts and key districts in which mass screening activities should be implemented have been identified. Efforts should also be made to scale up screening in border districts where HCVAb prevalence in the HIV population has been observed to be higher than average.

The main limitation of the present study is the absence of confirmation of chronic HCV infection with HCV RNA testing. Several studies have suggested that HCV serology is prone to false-positive results, especially in HIV-infected patients in SSA where the proportion of individuals confirmed by detection of HCV RNA after a positive HCVAb test is lower than other parts of the world [12, 30, 31]. Further, HCVAb presence includes those with resolved infection and as such does not necessarily confer active infection. There is conflicting evidence surrounding viremia rate across SSA, suggesting the possibility of localised outbreaks and the need for specific data to understand endemicity [12, 30, 32–34]. Molecular characterisation is required to determine the prevalence of chronic HCV and to accurately reflect current needs for HCV treatment in Rwanda. Despite this, the prevalence of HCVAb is still meaningful, filling knowledge gaps on individuals more likely to become infected with HCV.

This study was further limited by its retrospective nature. Ideally, this would have been an opportunity to collate risk factors for viral hepatitis infection in Rwanda. The authors decided against a risk factor regression analysis as many key risk factors for infection were not collected and the model would have been of limited application.

## Conclusions

This study adds to the understanding of viral hepatitis among HIV-infected individuals in Rwanda. The expanded viral hepatitis testing activity has achieved early diagnosis of individuals that will facilitate linkage to further testing and care, reducing hepatitis-related morbidity and curbing onward disease transmission. The findings presented should be used to inform policy and targeted screening activities, and should be extrapolated beyond the HIV positive population. Additional studies should be performed in HIV positive and HIV negative individuals to fill the remaining gaps in the epidemiological profile of these diseases and to determine risk factors for infection. In particular, molecular characterisation and detection of other markers should be conducted to determine the exact prevalence of viral hepatitis in this population.

## Abbreviations

ART: Antiretroviral therapy
CI: Confidence interval
ELISA: Enzyme-linked immunosorbent assay
HBsAg: Hepatitis B surface antigen
HBV: Hepatitis B
HCV: Hepatitis C
HCVAb: Hepatitis C antibody
HIV: Human immunodeficiency virus
RBC: Rwanda Biomedical Centre
SSA: Sub-Saharan Africa
